# A Comparative Study of Human TLR 7/8 Stimulatory Trimer Compositions in Influenza A Viral Genomes

**DOI:** 10.1371/journal.pone.0030751

**Published:** 2012-02-17

**Authors:** Chu-Wen Yang, Sy-Mien Chen

**Affiliations:** 1 Department of Microbiology, Soochow University, Shih-Lin, Taipei, Taiwan, Republic of China; 2 Department of Mathematics, Fu Jen Catholic University, Xinzhuang, New Taipei City, Taiwan, Republic of China; Hallym University, Republic of Korea

## Abstract

**Background:**

Variation in the genomes of single-stranded RNA viruses affects their infectivity and pathogenicity in two ways. First, viral genome sequence variations lead to changes in viral protein sequences and activities. Second, viral genome sequence variation produces diversity at the level of nucleotide composition and diversity in the interactions between viral RNAs and host toll-like receptors (TLRs). A viral genome-typing method based on this type of diversity has not yet been established.

**Methodology/Principal Findings:**

In this study, we propose a novel genomic trait called the “TLR stimulatory trimer composition” (TSTC) and two quantitative indicators, Score S and Score N, named “TLR stimulatory scores” (TSS). Using the complete genome sequences of 10,994 influenza A viruses (IAV) and 251 influenza B viruses, we show that TSTC analysis reveals the diversity of Score S and Score N among the IAVs isolated from various hosts. In addition, we show that low values of Score S are correlated with high pathogenicity and pandemic potential in IAVs. Finally, we use Score S and Score N to construct a logistic regression model to recognize IAV strains that are highly pathogenic or have high pandemic potential.

**Conclusions/Significance:**

Results from the TSTC analysis indicate that there are large differences between human and avian IAV genomes (except for segment 3), as illustrated by Score S. Moreover, segments 1, 2, 3 and 4 may be major determinants of the stimulatory activity exerted on human TLRs 7 and 8. We also find that a low Score S value is associated with high pathogenicity and pandemic potential in IAV. The *π* value from the TSS-derived logistic regression model is useful for recognizing emerging IAVs that have high pathogenicity and pandemic potential.

## Introduction

In single-stranded RNA viruses, genome sequence diversity affects infectiousness and pathogenicity in two ways. First, diversity in viral genome sequences leads to alternations of viral protein sequences and, consequently, changes in viral protein activity that may affect replication, transmission or antigenicity (interactions with the host's adaptive immunity). These issues have been extensively addressed by various phylogenetic and experimental studies of viral protein functions [1], [2], [3], [4].

The second way that genomic diversity affects infectiousness and pathogenicity is by affecting the strength of interactions between viral RNA and the innate immunity of the host. The avian and mammalian toll-like receptors (TLRs) 7 and 8 are usually present in the endosomal compartments, where they are responsible for detecting the single-stranded RNAs of viruses engulfed via endocytosis [5], [6], [7]. How TLRs 7 and 8 discriminate between self and non-self RNAs is not clear. However, published data indicate that nucleotide composition is crucial [8], [9], [10]. Diversity in viral genome sequences results in differences in nucleotide composition that may affect the stimulatory activity that viral RNAs exert on host TLRs. Genome sequence diversity may thus provide a way for single-stranded RNA viruses to evade host innate immunity. Very few attempts have been made to examine these types of virus-host interactions computationally.

In this study, we develop a computational method to evaluate the ability of single-stranded RNA virus genomes to stimulate TLRs 7/8 based on their nucleotide composition. We focus on stimulatory activity toward human TLRs (hTLRs) 7/8 because all of the TLRs 7/8 stimulatory oligoribonucleotide (ORN) sequences we examined were collected from literatures that used human cells as experimental models. We propose a novel genomic trait for single-stranded RNA viruses, called “TLR stimulatory trimer composition” (TSTC), which can be used to analyze the interactions between a single-stranded RNA virus genome and host TLRs 7/8. In this analysis, the frequencies of different nucleotide trimers found in the 96 hTLRs 7/8 stimulatory ORN sequences collected from literatures are calculated to construct a weight vector. If the relative frequency of a trimer in the hTLRs-7/8-stimulatory ORN sequences exceeds 1/64 (the expected value under a random distribution), we consider that trimer to be hTLRs-7/8-stimulatory. Otherwise the trimer is non-hTLRs-7/8-stimulatory. Each trimer is assigned a weight based on the logarithm of its relative frequency (see Methods section for details). For each viral genome analyzed, we determined the sum of weights of the hTLRs-7/8-stimulatory trimers (Score S) and the sum of weights of the non-hTLRs-7/8-stimulatory trimers (Score N) using the weights described above. These scores are called the “TLR stimulatory scores” (TSSs). Higher TSSs indicate that a greater number of trimers in the viral RNA genome are hTLRs-7/8-stimulatory, which implies a stronger interaction between the viral RNA and the host (human) TLRs 7/8. Conversely, lower TSSs indicate that a greater number of trimers in the viral RNA genome are non-hTLRs-7/8-stimulatory and exhibit a weaker interaction with the host (human) TLRs 7/8.

We use the influenza virus as an example in this study because a large number of influenza virus genome sequences are available in the NCBI Influenza database. Using the complete genome sequences of 10,994 influenza A viruses (IAV) and 251 influenza B viruses (IBV) from the NCBI Influenza Virus Resource, we demonstrate the diversity of TSSs among IAVs isolated from different hosts (human, avian and mammalian). Moreover, we illustrate the TSS differences between high- and low- pathogenicity IAVs. Finally, we use TSSs to construct a logistic regression model. We demonstrate that the *π* value computed from the logistic regression model for each IAV can be used to evaluate the probability of the virus having a high pathogenicity and pandemic potential.

## Results

### TSSs of IAV genomes from human hosts are higher than those of IAV genomes from avian hosts

**Figure 1:**
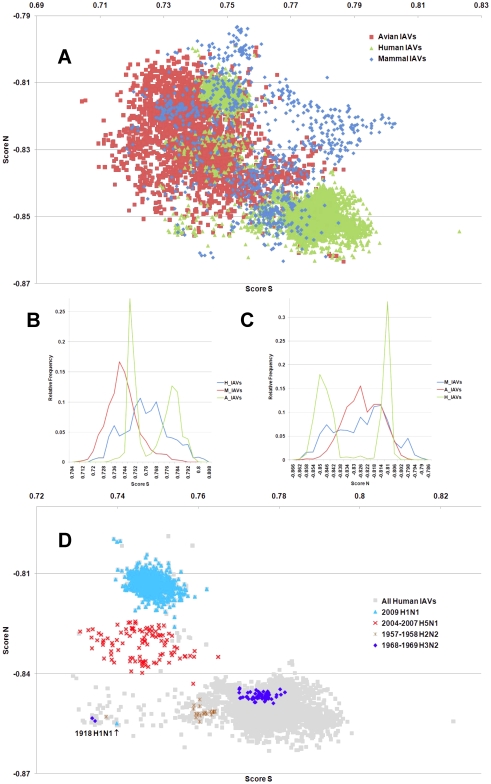
The hTLR stimulatory score distributions for segment 1. 10,994 IAVs, including 3,324 avian (red), 6,658 human (green) and 1,012 mammalian (blue) IAVs were used in this analysis. (A) The distribution of the hTLR stimulatory scores of segment 1 genomic RNAs. The x-axis represents Score S, the y-axis represents Score N. (B) The relative frequency distribution of Score S from segment 1 genomic RNAs. The x-axis indicates Score S, the y-axis gives the relative frequency. (C) The relative frequency distribution of Score N from segment 1 genomic RNAs. The x-axis represents Score N, the y-axis represents the relative frequency. (D) The distribution of the hTLR stimulatory scores of segment 1 genomic RNAs from 6,658 human IAVs (gray). The highly pathogenic/pandemic-associated IAVs are highlighted. The x-axis represents Score S, and the y-axis represents Score N.

**Figure 2 pone-0030751-g002:**
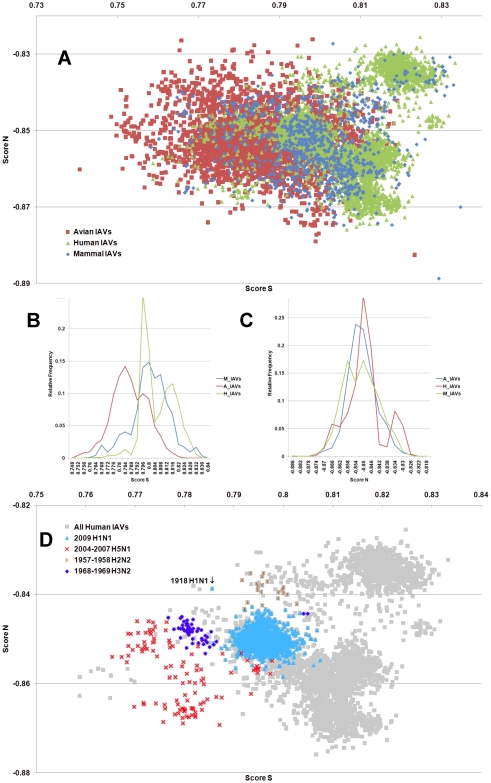
The hTLR stimulatory score distributions for segment 2. 10,994 IAVs, including 3,324 avian (red), 6,658 human (green) and 1,012 mammalian (blue) IAVs were used in this analysis. (A) The distributions of the hTLR stimulatory scores of segment 2 genomic RNAs. The x-axis represents Score S, the y-axis represents Score N. (B) The relative frequency distribution of Score S from segment 2 genomic RNAs. The x-axis indicates Score S, the y-axis gives the relative frequency. (C) The relative frequency distribution of Score N from segment 2 genomic RNAs. The x-axis represents Score N, the y-axis represents the relative frequency. (D) The distribution of the hTLR stimulatory scores of segment 2 genomic RNAs from 6,658 human IAVs (gray). The pandemic-associated IAVs are highlighted. The x-axis represents Score S, and the y-axis represents Score N.

**Figure 3 pone-0030751-g003:**
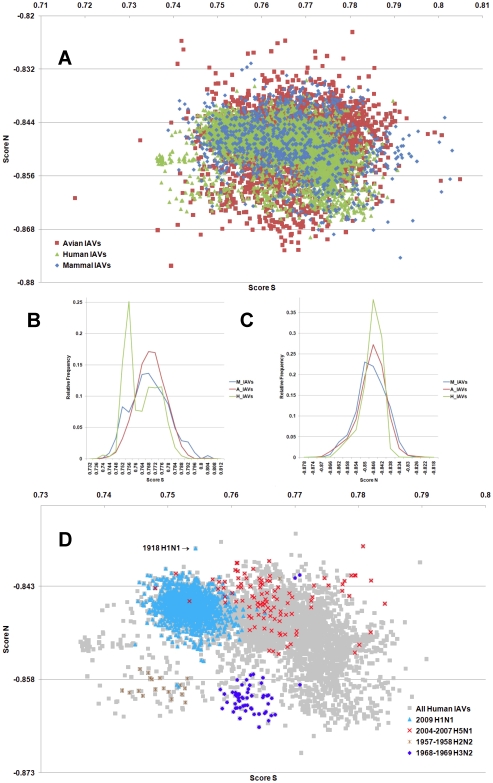
The hTLR stimulatory score distributions for segment 3. 10,994 IAVs, including 3,324 avian (red), 6,658 human (green) and 1,012 mammalian (blue) IAVs were used in this analysis. (A) The distributions of the hTLR stimulatory scores of segment 3 genomic RNAs. The x-axis represents Score S, the y-axis represents Score N. (B) The relative frequency distribution of Score S from segment 3 genomic RNAs. The x-axis indicates Score S, the y-axis gives the relative frequency. (C) The relative frequency distribution of Score N from segment 3 genomic RNAs. The x-axis represents Score N, the y-axis represents the relative frequency. (D) The distribution of the hTLR stimulatory scores of segment 3 genomic RNAs from 6,658 human IAVs (gray). The pandemic-associated IAVs are highlighted. The x-axis represents Score S, and the y-axis represents Score N.

**Figure 4 pone-0030751-g004:**
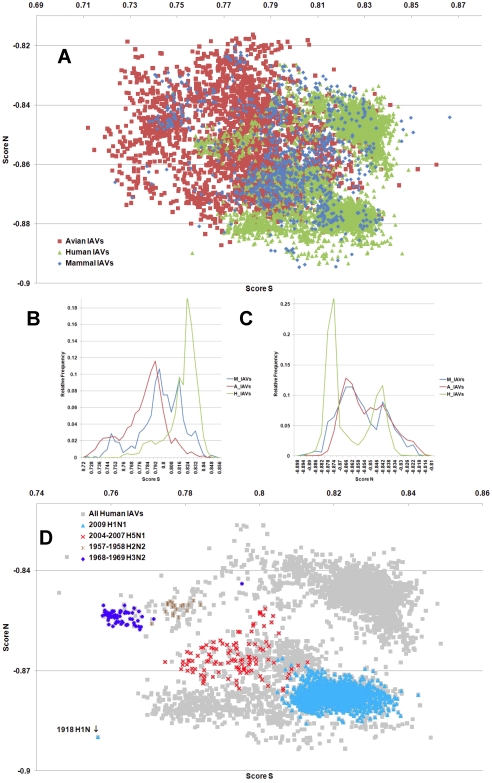
The hTLR stimulatory score distributions for segment 4. 10,994 IAVs, including 3,324 avian (red), 6,658 human (green) and 1,012 mammalian (blue) IAVs were used in this analysis. (A) The distributions of the hTLR stimulatory scores of segment 4 genomic RNAs. The x-axis represents Score S, the y-axis represents Score N. (B) The relative frequency distribution of Score S from segment 4 genomic RNAs. The x-axis indicates Score S, the y-axis gives the relative frequency. (C) The relative frequency distribution of Score N from segment 4 genomic RNAs. The x-axis represents Score N, the y-axis represents the relative frequency. (D) The distribution of the hTLR stimulatory scores of segment 4 genomic RNAs from 6,658 human IAVs (gray). The pandemic-associated IAVs are highlighted. The x-axis represents Score S, and the y-axis represents Score N.

**Figure 5 pone-0030751-g005:**
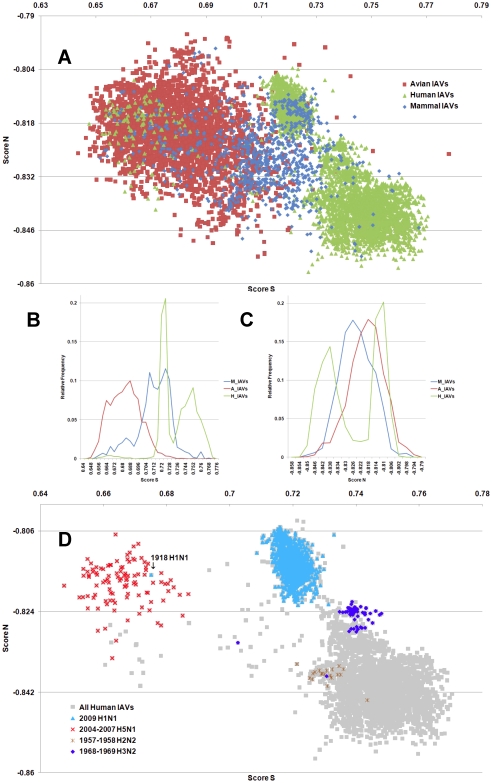
The hTLR stimulatory score distributions for segment 5. 10,994 IAVs, including 3,324 avian (red), 6,658 human (green) and 1,012 mammalian (blue) IAVs were used in this analysis. (A) The distributions of the hTLR stimulatory scores of segment 5 genomic RNAs. The x-axis represents Score S, the y-axis represents Score N. (B) The relative frequency distribution of Score S from segment 5 genomic RNAs. The x-axis indicates Score S, the y-axis gives the relative frequency. (C) The relative frequency distribution of Score N from segment 5 genomic RNAs. The x-axis represents Score N, the y-axis represents the relative frequency. (D) The distribution of the hTLR stimulatory scores of segment 5 genomic RNAs from 6,658 human IAVs (gray). The pandemic-associated IAVs are highlighted. The x-axis represents Score S, and the y-axis represents Score N.

**Figure 6 pone-0030751-g006:**
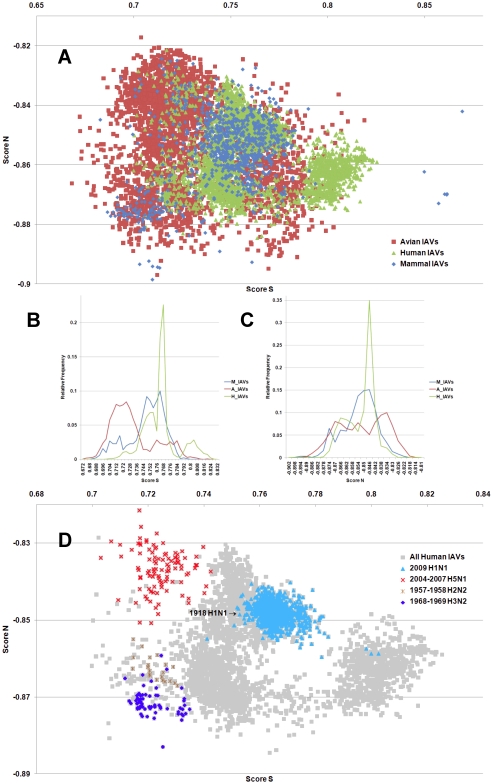
The hTLR stimulatory score distributions for segment 6. 10,994 IAVs, including 3,324 avian (red), 6,658 human (green) and 1,012 mammalian (blue) IAVs were used in this analysis. (A) The distributions of the hTLR stimulatory scores of segment 6 genomic RNAs. The x-axis represents Score S, the y-axis represents Score N. (B) The relative frequency distribution of Score S from segment 6 genomic RNAs. The x-axis indicates Score S, the y-axis gives the relative frequency. (C) The relative frequency distribution of Score N from segment 6 genomic RNAs. The x-axis represents Score N, the y-axis represents the relative frequency. (D) The distribution of the hTLR stimulatory scores of segment 6 genomic RNAs from 6,658 human IAVs (gray). The pandemic-associated IAVs are highlighted. The x-axis represents Score S, and the y-axis represents Score N.

**Figure 7 pone-0030751-g007:**
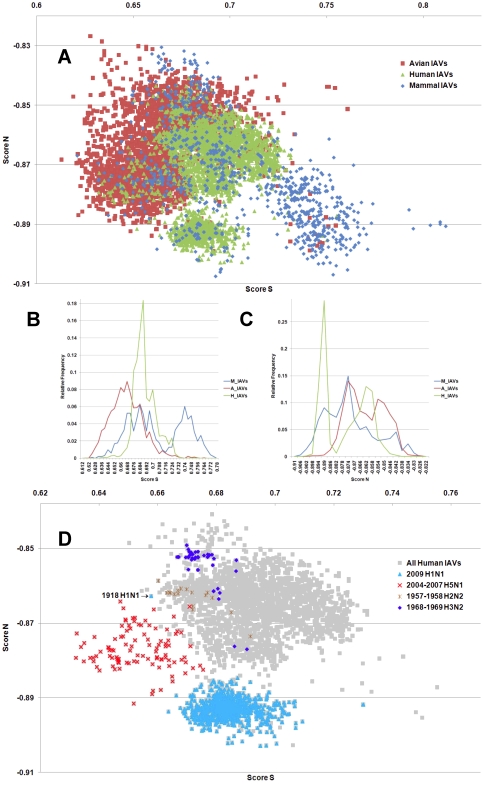
The hTLR stimulatory score distributions for segment 7. 10,994 IAVs, including 3,324 avian (red), 6,658 human (green) and 1,012 mammalian (blue) IAVs were used in this analysis. (A) The distributions of the hTLR stimulatory scores of segment 7 genomic RNAs. The x-axis represents Score S, the y-axis represents Score N. (B) The relative frequency distribution of Score S from segment 7 genomic RNAs. The x-axis indicates Score S, the y-axis gives the relative frequency. (C) The relative frequency distribution of Score N from segment 7 genomic RNAs. The x-axis represents Score N, the y-axis represents the relative frequency. (D) The distribution of the hTLR stimulatory scores of segment 7 genomic RNAs from 6,658 human IAVs (gray). The pandemic-associated IAVs are highlighted. The x-axis represents Score S, and the y-axis represents Score N.

**Figure 8 pone-0030751-g008:**
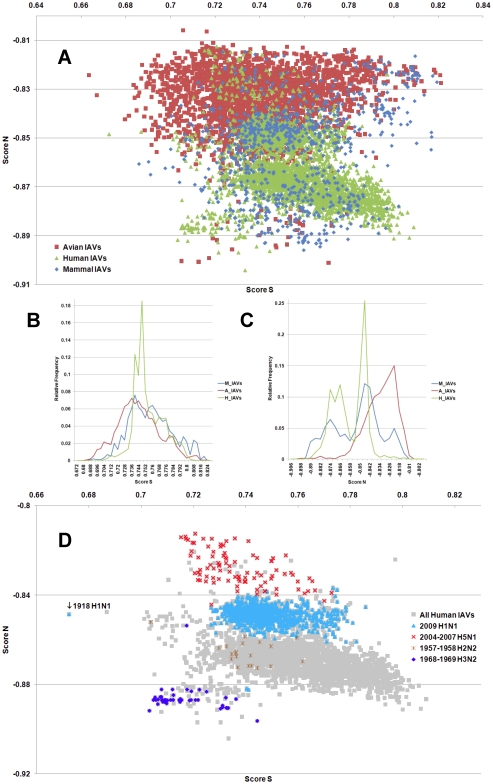
The hTLR stimulatory score distributions for segment 8. 10,994 IAVs, including 3,324 avian (red), 6,658 human (green) and 1,012 mammalian (blue) IAVs were used in this analysis. (A) The distributions of the hTLR stimulatory scores of segment 8 genomic RNAs. The x-axis represents Score S, the y-axis represents Score N. (B) The relative frequency distribution of Score S from segment 8 genomic RNAs. The x-axis indicates Score S, the y-axis gives the relative frequency. (C) The relative frequency distribution of Score N from segment 8 genomic RNAs. The x-axis represents Score N, the y-axis represents the relative frequency. (D) The distribution of the hTLR stimulatory scores of segment 8 genomic RNAs from 6,658 human IAVs (gray). The pandemic-associated IAVs are highlighted. The x-axis represents Score S, and the y-axis represents Score N.

**Figure 9:**
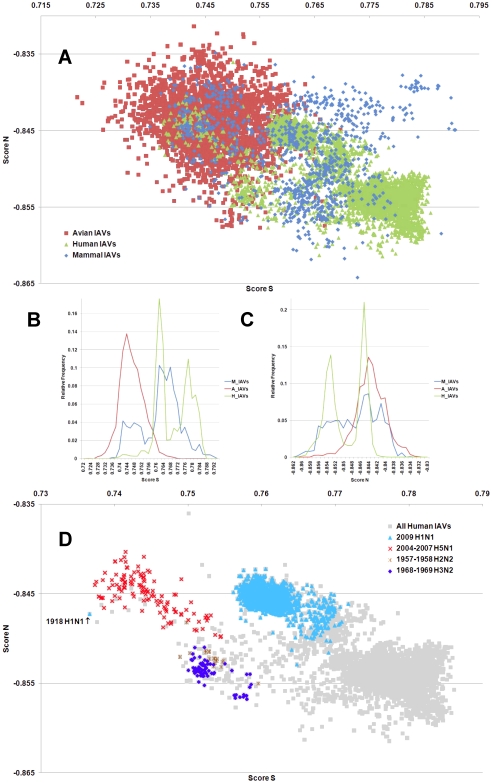
The hTLR stimulatory score distributions of the whole genome (all eight segments combined). 10,994 IAVs, including 3,324 avian (red), 6,658 human (green) and 1,012 mammalian (blue) IAVs were used in this analysis. (A) The distributions of the hTLR stimulatory scores from eight genomic RNAs. The x-axis represents Score S; the y-axis represents Score N. (B) The relative frequency distributions of Score S from eight genomic RNAs. The x-axis represents Score S; the y-axis represents the relative frequency. (C) The relative frequency distributions of Score N from eight genomic RNAs. The x-axis represents Score N; the y-axis represents the relative frequency. (D) The distribution of the hTLR stimulatory scores from eight genomic RNAs from 6658 human IAVs (gray). The pandemic-associated IAVs are highlighted. The x-axis represents Score S, and the y-axis represents Score N.

**Figure 10:**
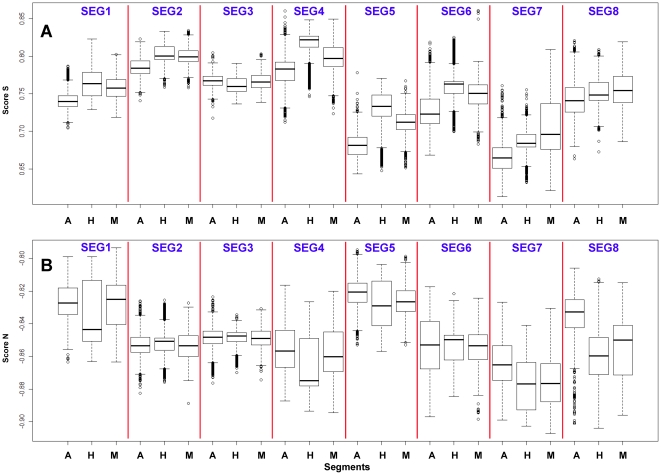
The hTLR stimulatory score distribution of eight segments of IAVs from different hosts. 10,994 IAVs, including 3,324 avian [A], 6,658 human [H] and 1,012 mammalian [M] IAVs were used in this analysis. (A) The distribution of Score S for eight genomic RNAs. The x-axis shows the different host species, and the y-axis represents Score S. (B) The distribution of Score N for eight genomic RNAs. The x-axis represents the hosts, and the y-axis represents Score N.

**Figure 11:**
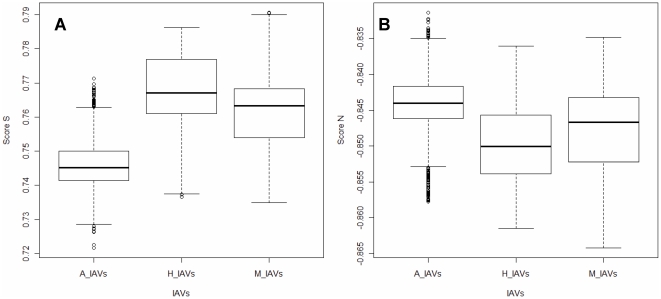
The hTLR stimulatory score distribution of IAVs from different hosts. A total of 10,994 IAV genomes, including 3,324 avian, 6,658 human and 1,012 mammalian IAVs were used in this analysis. For each IAV, eight segments were combined to compute the TLR stimulatory scores: (A) Score S and (B) Score N. The x-axis is the IAVs from different hosts (A_IAVs from avian, H_IAVs from human and M_IAVs from mammal); the y-axis is the TLR stimulatory score (Score S for left, Score N for right).

IAV is a zoonotic virus. To understand whether there are significant differences between the IAV genomes from different hosts, we compared the TSS distributions (as defined in the methods section) of avian, human and mammalian IAVs. The TSS distributions of the IAV genomic RNA segments 1–8 are shown in Figures 1, 2, 3, 4, 5, 6, 7, 8. The TSS distribution of whole genome (a combination of all eight segments) is illustrated in Figure 9. As shown in Figures 1A–[Fig pone-0030751-g002] [Fig pone-0030751-g003] [Fig pone-0030751-g004] [Fig pone-0030751-g005] [Fig pone-0030751-g006] [Fig pone-0030751-g007] 8A, the areas covered by the TSSs of human IAVs on the Score S-Score N plane are largely distinct from the areas covered by the TSSs of avian IAVs, except for segment 3. Moreover, the majority of TSSs from avian IAVs fall within the lower Score S and higher Score N areas, except for segment 3 (Figures 1A–[Fig pone-0030751-g002] [Fig pone-0030751-g003] [Fig pone-0030751-g004] [Fig pone-0030751-g005] [Fig pone-0030751-g006] [Fig pone-0030751-g007] 8A, 1B–[Fig pone-0030751-g002] [Fig pone-0030751-g003] [Fig pone-0030751-g004] [Fig pone-0030751-g005] [Fig pone-0030751-g006] [Fig pone-0030751-g007] 8B, 1C–[Fig pone-0030751-g002] [Fig pone-0030751-g003] [Fig pone-0030751-g004] [Fig pone-0030751-g005] [Fig pone-0030751-g006] [Fig pone-0030751-g007] 8C and Figures 10). The TSSs computed from the combined eight segments give similar results (Figures 9A, 9B, 9C and 11). These results imply that newly emerging IAVs from avian hosts might have lower hTLR 7/8 stimulatory activity. Conversely, IAVs that have adapted to human hosts have higher hTLR 7/8 stimulatory activity. Among the eight segments, segments 2 and 4 have the highest hTLR 7/8 stimulatory activity, followed by segments 1 and 3 (Figure 10). Therefore, the trimer compositions of these four segments may be the major determinants of hTLR 7/8 evasion.

Another interesting phenomenon is that the patterns of TSS distributions of IAVs from avian hosts are distinct from those of IAVs from human hosts. The TSS distributions of IAVs from avian hosts have only one group for each segment (the red regions in Figures 1A–[Fig pone-0030751-g002] [Fig pone-0030751-g003] [Fig pone-0030751-g004] [Fig pone-0030751-g005] [Fig pone-0030751-g006] [Fig pone-0030751-g007] 8A, 1B–[Fig pone-0030751-g002] [Fig pone-0030751-g003] [Fig pone-0030751-g004] [Fig pone-0030751-g005] [Fig pone-0030751-g006] [Fig pone-0030751-g007] 8B and 1C–[Fig pone-0030751-g002] [Fig pone-0030751-g003] [Fig pone-0030751-g004] [Fig pone-0030751-g005] [Fig pone-0030751-g006] [Fig pone-0030751-g007] 8C). In contrast, the TSS distributions of IAVs from human hosts display separate clusters except for segment 3 (the green regions in Figures 1A–[Fig pone-0030751-g002] [Fig pone-0030751-g003] [Fig pone-0030751-g004] [Fig pone-0030751-g005] [Fig pone-0030751-g006] [Fig pone-0030751-g007] 8A, 1B–[Fig pone-0030751-g002] [Fig pone-0030751-g003] [Fig pone-0030751-g004] [Fig pone-0030751-g005] [Fig pone-0030751-g006] [Fig pone-0030751-g007] 8B, 1C–[Fig pone-0030751-g002] [Fig pone-0030751-g003] [Fig pone-0030751-g004] [Fig pone-0030751-g005] [Fig pone-0030751-g006] [Fig pone-0030751-g007] 8C and all colors in Figures 1D–[Fig pone-0030751-g002] [Fig pone-0030751-g003] [Fig pone-0030751-g004] [Fig pone-0030751-g005] [Fig pone-0030751-g006] [Fig pone-0030751-g007] 8D). These differences in score distribution patterns might indicate something interesting that is worthy of further investigations.

### TSSs of highly pathogenic/pandemic human IAVs form specific subsets

To explore the TSSs distribution differences between high pathogenicity/pandemic potential and other human IAVs, the TSSs from high pathogenicity/pandemic potential IAVs were highlighted on the TSS distribution map of 6,658 human IAVs for each segment (Figures 1D–[Fig pone-0030751-g002] [Fig pone-0030751-g003] [Fig pone-0030751-g004] [Fig pone-0030751-g005] [Fig pone-0030751-g006] [Fig pone-0030751-g007] 8D) and for the whole genome (Figure 9D). In Figures 1–8, two interesting features stand out. First, most of TSSs from high pathogenicity/pandemic potential IAVs fall within the lower Score S areas, except for segments 4 and 8. Because TSSs are designed to be indicators for hTLRs 7/8 stimulatory activity, these results indicate that low hTLRs stimulatory activity (corresponding to an ability to evade TLRs 7/8) is favoured for high pathogenicity/pandemic potential IAVs. This phenomenon is significant, especially for H5N1, in which five of the eight segments (segments 1, 2, 5, 6, and 7) have the lowest Score S. Second, the TSSs from most of the H5N1 IAVs (red crosses in Figures 1D–[Fig pone-0030751-g002] [Fig pone-0030751-g003] [Fig pone-0030751-g004] [Fig pone-0030751-g005] [Fig pone-0030751-g006] [Fig pone-0030751-g007] 8D), especially segments 1, 2, 5, 6, 7 and 8, are separated from the main body of human IAV TSSs (gray regions in Figures 1D–[Fig pone-0030751-g002] [Fig pone-0030751-g003] [Fig pone-0030751-g004] [Fig pone-0030751-g005] [Fig pone-0030751-g006] [Fig pone-0030751-g007] 8D) but are within the areas covered by the avian IAV TSSs (the red regions in Figures 1A–[Fig pone-0030751-g002] [Fig pone-0030751-g003] [Fig pone-0030751-g004] [Fig pone-0030751-g005] [Fig pone-0030751-g006] [Fig pone-0030751-g007] 8A). These results might indicate that H5N1 IAVs isolated from humans still retain the characteristics of avian IAVs that distinguish them from human H1N1 and H3N2 IAVs.

### The relationship between TSSs and pathogenicity/pandemic potential

**Figure 12:**
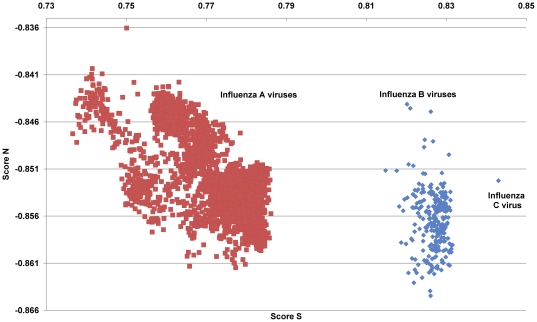
The hTLR stimulatory score distributions of the three types of influenza viruses. The values of the TLR stimulatory score, Score S and Score N, of the human influenza A (6,658 genomes), B (251 genomes) and C (1 genome) viral genomes are illustrated.

It is interesting that TSSs of the 2,947 pandemic/highly pathogenic IAVs on the TSS distribution map of 6,658 human IAVs indicate that the TSSs of those viruses are clustered in the low Score S area (Figure 9D). To establish the relationship between the TSSs and IAV pathogenicity/pandemic potential, an analysis of 6,658 influenza A genomes, 251 influenza B genomes and 1 influenza C genomes was performed. As shown in Figure 12, the type C virus has the highest Score S, and the type A viruses have the lowest Score S values. This result is consistent with the fact that pathogenicity/pandemic potential of the three types of influenza viruses follows the trend A>B>C, and it suggests that TSSs may be useful as indicators of pathogenicity and pandemic potential of influenza viruses.

### Displaying viral dynamics using *π* values from a logistic regression model

To establish a model for the prediction of pathogenicity and pandemic potential using TSSs, the 10-fold cross-validation method was used. Details of model construction and the selection procedure are described in the methods section. Briefly, based on 100 re-sampling iterations and 10-fold cross-validation, a model with coefficients derived from the averaged coefficients of all models that pass the goodness of fit test was established and used for the prediction of viral pathogenicity and pandemic potential. Using *π* to denote the probability that an IAV has high pathogenicity/pandemic potential, we have log (*π*/(1−*π*)) = −6.83–0.74 ⋅ Score S_1_+7.11 ⋅ Score N_1_−43.1 ⋅ Score S_2_−68.63 ⋅ Score N_2_−105.01 ⋅ Score S_3_−138.05 ⋅ Score N_3_−105.51 ⋅ Score S_4_−21.16 ⋅ Score N_4_+1.04 ⋅ Score S_5_+81.75 ⋅ Score N_5_−51.83 ⋅ Score S_6_+89.45 ⋅ Score N_6_+5.9 ⋅ Score S_7_−233.07 ⋅ Score N_7_−35.4 ⋅ Score S_8_−20.88 ⋅ Score N_8_.

**Figure 13:**
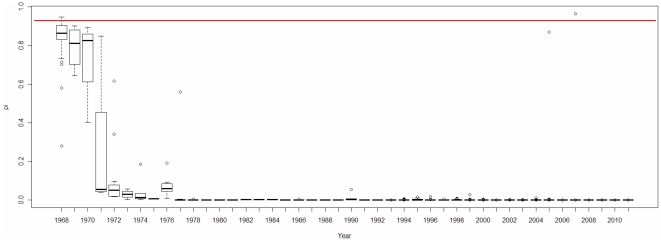
Yearly dynamics of the human H3N2 IAVs displayed by the *π* value from the logistic regression model. A box plot is used to illustrate the yearly dynamics of 2,096 human H3N2 IAVs from the IAV database. The x-axis gives the years in which the IAV data were recorded, while the y-axis indicates the *π* values of the IAVs, as computed by the logistic regression model. The red line indicates *π* = 0.95.

**Figure 14:**
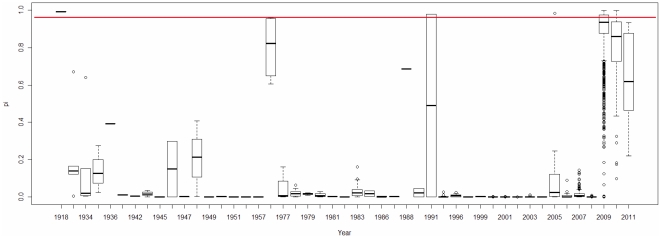
Yearly dynamics of the human H1N1 IAVs displayed by the *π* value from the logistic regression model. A box plot is used to illustrate the yearly dynamics of 3,764 human H1N1 IAVs from the IAV database. The x-axis indicates the year in the record of the IAV data, and the y-axis indicates the *π* values of the IAVs as computed by the logistic regression model. The red line indicates *π* = 0.95.

**Figure 15:**
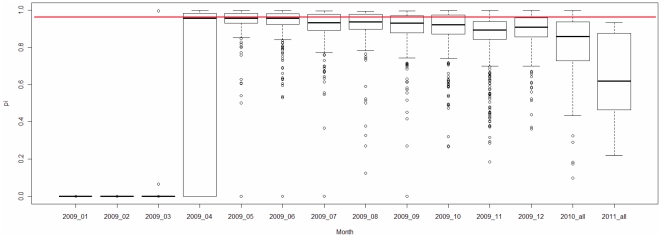
Monthly dynamics of the human H1N1 IAVs (2009–2010) displayed by the *π* value from the logistic regression model. A box plot is used to illustrate the monthly dynamics of 2,851 2009–2011 H1N1 IAVs from the IAV database. The x-axis indicates the month of each IAV data record, and the y-axis gives the *π* values of the IAVs as computed by the logistic regression model. The red line indicates *π* = 0.95.

Based on the regression model obtained, we computed the *π* values of the human H3N2 and H1N1 IAVs in the database and displayed the distributions of the *π* values by years (Figures 13, 14) and months (Figure 15). The *π* value indicates the probability of each IAV having a high pathogenicity/pandemic potential. Taking H3N2 as an example, we find that pandemic H3N2 IAVs (*π*>0.95 in Figure 13) appeared in 1968 and completely disappeared from 1972 to 2009. Notably, human H3N2 IAV strain with a high *π* value (*π*>0.95 in Figure 13) emerged in 2007. Fortunately, this H3N2 IAV strain did not cause a pandemic, possibly because H3N2 antibodies were already present in human population. Human H1N1 IAV strains with high *π* values (*π*>0.95 in Figure 14) appeared more frequently than high-*π*-value H3N2 IAVs. They appeared in 1918, 1976, 1991, 2005, 2009 and 2010. These results are consistent with the history of H1N1 outbreaks (e.g., the 1918 Spanish H1N1 pandemic, the 1977–1978 Russian H1N1 outbreak and the 2009 H1N1 pandemic) [7]. The 1991 and 2005 high-*π*-value strains did not cause pandemics, possibly because H1N1 antibodies were already present in human population. The large amount of data on the 2009 H1N1 IAVs provided detailed information on the viral dynamics of the 2009 pandemic. The *π* values of the 2009 H1N1 IAVs, grouped by month, indicate that a pandemic strain first appeared in March 2009 (Figure 15). High-*π*-value (*π*>0.95) strains persisted until 2010. These results indicate that the *π* value computed from the TSS-based logistic regression model is useful for the surveillance of IAVs with a high pathogenicity/pandemic potential.

## Discussion

It is well known that the NS1 protein is an immunosuppressor. It inhibits innate immunity by preventing type I IFN release, and it inhibits adaptive immunity by attenuating human DC maturation and reducing the capacity of DCs to induce a T-cell response [11], [12]. However, the effects of NS1 occur after successful infection, viral RNA transcription and viral protein production. Before viral RNA transcription and protein production can take place, single-stranded RNA viruses must first conquer another innate immune mechanism: the toll-like receptors 7 and 8 of the host cells [5], [6], [7].

To evaluate the diversity of interactions between viral genomic RNAs and host TLR 7/8, we devise a novel viral genomic trait called TSTC and derive two scores called TSSs. A comparison of the TSS distributions from each genomic RNA (Figures 1–8) and from the whole genomes (Figure 9) of human, avian and mammalian IAVs revealed that there are large differences between human and avian IAV genomes, as indicated by Score S and Score N (Figures 1A–[Fig pone-0030751-g002] [Fig pone-0030751-g003] [Fig pone-0030751-g004] [Fig pone-0030751-g005] [Fig pone-0030751-g006] [Fig pone-0030751-g007] 8A, 1B–[Fig pone-0030751-g002] [Fig pone-0030751-g003] [Fig pone-0030751-g004] [Fig pone-0030751-g005] [Fig pone-0030751-g006] [Fig pone-0030751-g007] 8B, 1C–[Fig pone-0030751-g002] [Fig pone-0030751-g003] [Fig pone-0030751-g004] [Fig pone-0030751-g005] [Fig pone-0030751-g006] [Fig pone-0030751-g007] 8C, 10 and 11), except for segment 3. Moreover, we found that a low Score S is associated with high pathogenicity/pandemic potential of IAVs (Figures 1D–[Fig pone-0030751-g002] [Fig pone-0030751-g003] [Fig pone-0030751-g004] [Fig pone-0030751-g005] [Fig pone-0030751-g006] [Fig pone-0030751-g007] [Fig pone-0030751-g008] 9D and 12).

The algorithm proposed in this study was based on the identified TLR stimulatory activities of naked synthetic oligos. However, Influenza viruses are enveloped and negative-sensed RNA viruses and the virus genomes are composed of ribonucleoproteins instead of naked RNAs. An interesting question raised will be “How can an algorithm derived from naked oligoribonucleotides used to analyze the TLR stimulatory properties of the viral RNPs”. To answer this question, we have to look back the discovery of the natural ligands of TLR 7. TLR7 was demonstrated to mediate pDC responses to ssRNA viruses such as influenza, vesicular stomatitis virus, and Sendai virus [13], [14]. Viral genomic ssRNA purified from influenza virions or synthetic ssRNA oligoribonucleotides containing U or GU repeats can substitute for intact influenza in triggering IFN-α production by pDC cells [14], [15]. Moreover, the acidification of endosome is essential for viral ssRNA recognition by pDC cells [13], [16]. These observations led to a model proposed by Heil et al. in which viruses are taken up by pDCs (or other cells) and are subjected to proteolytic degradation in the endosomal compartment, exposing their RNA genomes for recognition by TLR7 [15]. Using vesicular stomatitis virus Lee et al. demonstrated that viral ssRNA in cytoplasm can be transported into endosome through autophagy pathway [17]. Although the matrix protein 2 of IAV blocks autophagosome fusion with lysosomes to prevent host cell apoptosis [18], [19], inhibition of autophagy leads to decrease of IAV replication [20]. Therefore, autophagy pathway is active in IAV infected host cells and might be the second route to deliver the cytoplasmic IAV RNA to the endosome for recognition by TLR 7/8. Together, these results indicate that TLR 7/8 interacts with naked viral ssRNAs from different routes. Therefore, synthetic oligoribonucleotides are commonly used as ligands for studies of TLR7/8 activity in several studies including the 9 papers we chosen. Evaluation of TLR 7 stimulatory activity of viral genomic RNAs based on the nucleotide frequency of naked synthetic TLR stimulatory oligos is feasible.

The *π* values from the TSS-derived logistic regression model are useful for recognizing emerging IAVs that have high pathogenicity/pandemic potential (Figures 13, 14 and 15). The advantage of using *π* is that *π* is a probability that can be used to evaluate the confidence of a prediction that an IAV has a high pathogenicity/pandemic potential. However, many factors can affect the pathogenicity/pandemic potential of an IAV, including 627K and 701N mutations of PB2 [21], [22], PB1-F2 [23], the multiple basic cleavage sites of HA [24], 92E mutation of NS1 [25] and the C-terminal ESEV motif of NS1 [26], [27]. The *π* value proposed in this study is an additional indicator that is helpful for characterizing viral pathogenicity/pandemic potential. High pathogenicity/pandemic potential IAVs have a high *π* value (*π*>0.95), but IAVs of high *π* value (*π*>0.95) do not necessarily have high pathogenicity/pandemic potential.

TSTC analysis requires complete viral genome sequences (including all eight segments), and it therefore can be costly to acquire the necessary data. Nevertheless, rapid improvements and the automation of sequencing will make this method more feasible in the future. TSTC analysis provides a new option for data analysis in large-scale sequencing-based approaches, such as metagenomic projects involving IAVs [28].

**Figure 16:**
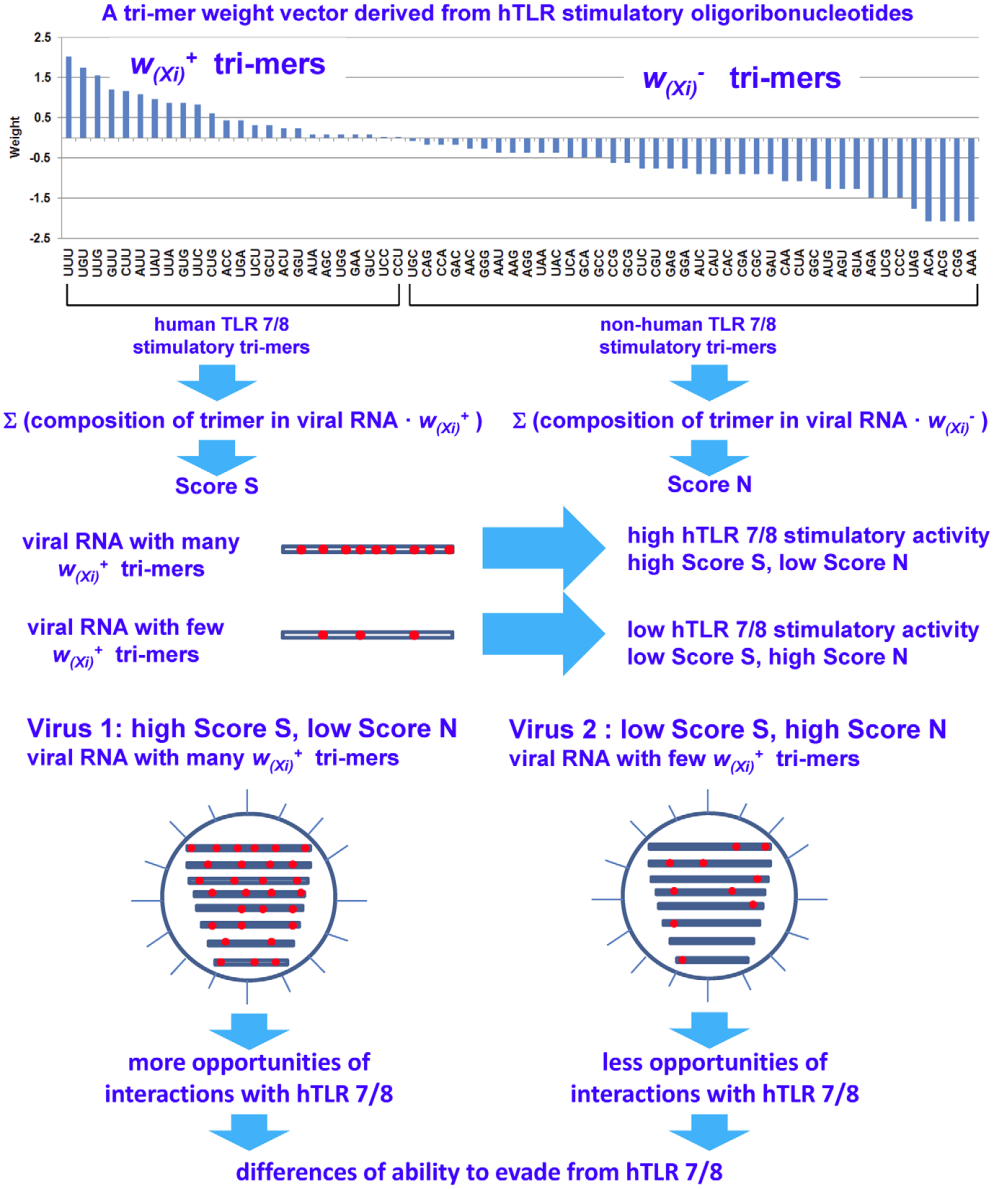
Method design for scoring the interactions between viral RNAs and host (human) toll-like receptors 7/8 by analyzing the TLR 7/8 stimulatory trimer composition of viral RNAs.

In this study, a novel viral genomic trait, “TLR stimulatory trimer composition” (TSTC), was proposed (Figure 16). A comparison of the TSS distributions of eight genomic RNAs and whole genomes of human, avian and mammalian IAVs revealed the following: (1) There are large differences between human and avian IAV genomes, as indicated by their hTLRs 7/8 stimulatory trimer compositions (Score S, excluding segment 3; Figures 1A–[Fig pone-0030751-g002] [Fig pone-0030751-g003] [Fig pone-0030751-g004] [Fig pone-0030751-g005] [Fig pone-0030751-g006] [Fig pone-0030751-g007] 8A, 1B–[Fig pone-0030751-g002] [Fig pone-0030751-g003] [Fig pone-0030751-g004] [Fig pone-0030751-g005] [Fig pone-0030751-g006] [Fig pone-0030751-g007] 8B, 1C–[Fig pone-0030751-g002] [Fig pone-0030751-g003] [Fig pone-0030751-g004] [Fig pone-0030751-g005] [Fig pone-0030751-g006] [Fig pone-0030751-g007] 8C, 10 and 11). (2) Segment 4 has the highest hTLRs 7/8 stimulatory trimer composition, followed by segments 2, 1 and 3 (Figure 10). These four segments may be the major determinants of hTLRs 7/8 stimulatory activity for an IAV genome. (3) Low Score S values are associated with high pathogenicity/pandemic potential of IAVs (Figures 1D–[Fig pone-0030751-g002] [Fig pone-0030751-g003] [Fig pone-0030751-g004] [Fig pone-0030751-g005] [Fig pone-0030751-g006] [Fig pone-0030751-g007] [Fig pone-0030751-g008] 9D and 12). The *π* value from the TSS-derived logistic regression model is useful for recognizing emerging IAVs that have high pathogenicity/pandemic potential (Figures 13, 14 and 15).

## Materials and Methods

### Method design

To compare the diversity of hTLR 7/8 stimulatory activity among viral genomes, we began by uncovering the trinucleotide composition of hTLR 7/8 stimulatory ORNs at first. Then the difference of hTLR 7/8 stimulatory activity among viral genomes can be analyzed by computing the number of overrepresented and underrepresented trimers from hTLR 7/8 stimulatory ORNs in each viral genome RNA (or in the whole genome). Since the identity of human and mouse TLR 7 protein sequences is only 81%, the preferences of ligand nucleotide compositions of the two TLRs might be different. To determine the tri-nucleotide frequency of hTLR 7/8 stimulatory ORNs, two criteria were used for ORN sequences selection in this study. First, the assays of the human TLR7/8 stimulatory activity of the ORNs were performed on human PBMC or human primary pDC, monocytes isolated from PBMC. Second, the assays of the human TLR7/8 stimulatory activity were based on interferon and cytokine (IFNα, TNFα or IL12) productions which were detected by ELISA. We collected the sequences of 96 ORNs (57 oligonucleotides and 39 tetramers) that were experimentally validated as having stimulatory activity toward human TLRs 7/8 from nine papers. These ORN sequences and their corresponding references are listed in Table S1. Because the experiments validating the TLRs 7/8 stimulatory activity of these 96 ORN sequences were conducted using human cells, the TSTC and TSSs described in this study should be considered human specific. However, the same methods could be applied to other hosts, provided that information about TLR 7/8 stimulatory ORN sequences is available.

### Scoring scheme for the human TLR 7/8 stimulatory trimer composition of viral genomes

We devise a novel genetic trait, which we call “TLR 7/8 stimulatory trimer composition” (TSTC), and we use it to analyze the hTLRs 7/8 stimulatory activity of each viral genome. First of all, we compute the frequencies of hTLRs 7/8 stimulatory trimers in 96 ORNs to construct a weight vector (Figure 16).

Let the 4^3^ = 64 possible trimers be labeled as *X_1_*, *X_2_*, …, *X_64_*. Each trimer frequency 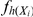 is defined as
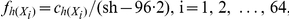
where 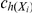 is the number of times the trimer, *X_i_*, appears among the 96 hTLR 7/8 stimulatory ORN sequences, and *s_h_* is the total length of the 96 hTLR 7/8 stimulatory ORN sequences. The denominator may be thought of as the number of possible positions for the first nucleotide in the trimer. Note that the trimer cannot start from the last two positions of the sequence. Then we construct a trimer weight vector, in which the value for the *i^th^* coordinate is given by
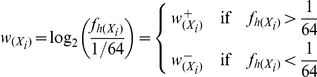
for *i* = 1, 2, …, 64. Note that 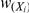 is positive when *X_i_* appears more frequently than expected in a completely random sample and is negative when *X_i_* appears less frequently than expected. We define weights for the overrepresented trimers as 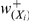 = 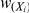 if 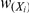 is positive, and define weights for the underrepresented trimers as 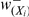 = 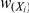 if 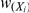 is negative. Trimers with positive weights were considered to be hTLRs-7/8-stimulatory. In contrast, trimers with negative weights were considered as non-hTLRs-7/8-stimulatory.

Given a RNA virus genome, we compute the positive and negative weighted trimer compositions which are referred to as Score S and Score N, respectively, and collectively referred to as “TLR 7/8 stimulatory scores” (TSSs). Score S for stimulatory trimers are calculated as
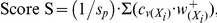
where 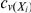 is the number of times that the trimer *X_i_* appears in the viral genomic RNA (with *i* = 1, 2, …, 64) and *s_p_* is the number of trimers with a positive weight. Similarly, Score N of non-hTLR-7/8-stimulatory trimers are calculated as
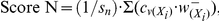
where 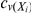 is the number of times that the trimer *X_i_* appears in the viral genomic RNA (with *i* = 1, 2, …, 64) and *s_n_* is the number of trimers with a negative weight. A high TSS indicates a greater number of trimers in the viral genome are hTLRs-7/8-stimulatory. A low TSS indicates a greater number of trimers in the viral RNAs that are non-hTLRs-7/8-stimulatory.

### Sequences of influenza virus genomes

The sequences of 10,994 complete influenza viral genomes were retrieved from the NCBI Influenza database (genome set 2011.7.29). The genome set was composed of 3,324, 6,658 and 1,012 complete IAV genomes (8 segments) isolated from avian, human and mammalian hosts, respectively, together with 251 complete influenza B virus genomes (8 segments). One set of complete influenza C virus genome sequences (7 segments) was also retrieved from the NCBI viral genome database. The negative-sensed genomic RNA sequences were used in this study because negative-sensed genomic RNAs of IAVs are expected to interact with hTLR 7/8 within endosomes. All the sequences retrieved from the NCBI Influenza database are positive-sensed and were converted into negative-sensed sequences by a Perl script written by the first author.

### Data sets of IAVs with high pathogenicity/pandemic potential

A subset of 2,947 human IAV genomes that appeared in historic pandemic periods was used to represent the “highly pathogenic” IAVs. The pandemics included the 1957–1958 Asian Flu (H2N2 from China, Singapore and Japan), the 1968–1969 Hong Kong Flu (H3N2 from Hong Kong), the 2009 H1N1 Swine Flu and the 1918 H1N1 Spanish Flu. In addition, the 2003–2005 H5N1 from Vietnam and Thailand, the 2005–2008 H5N1 viruses from Indonesia were also included. These highly pathogenic/pandemic IAVs are listed in Table S2. They were used as a positive data set (highly pathogenic/pandemic IAVs) for logistic regression. The other 3,180 human H2N2, H3N2, H1N1 and H5N1 IAVs not included in the positive data set were used as the negative data set for logistic regression (Table S3).

### The logistic regression model

To study the ability of TSSs to predict IAV pathogenicity/pandemic potential, a logistic regression model was constructed as follows:
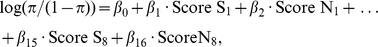
where *π* is the probability that an IAV has high pathogenicity/pandemic potential given the explanatory variables Score S*_i_* and Score N*_i_*, *i* = 1, …, 8; *β_0_* is the intercept term and *β_1_*, *β_2_* …*β_16_* are regression coefficients. A cross-validation method was used to evaluate the performance of the model and to find estimates of *β_1_*, *β_2_* …*β_16_* [29]. In this study, 90% of the entire data set is trained for a logistic regression model, and then tested on the rest 10% data. The maximum likelihood method was used to estimate the unknown parameters *β_1_*, *β_2_* …*β_16_* for each model. Model fitness was assessed by the Hosmer-Lemeshow goodness-of-fit test (H-L test). Model evaluation resulted in an H-L *p*-value>0.05 indicating a good fit. The cross-validation process is repeated 100 times using different splits of the data, which provides a good Monte-Carlo estimate of the complete cross-validation. In this study, 44 of 100 logistic regression models passed the H-L test. The performances of these 44 logistic regression models are listed in Table S4. The average of the intercepts from the 44 models is used as the intercept term in the final model. The rest of the 16 coefficients are computed in the same way, which leads to the final logistic regression model: *Y* = log (*π*/(1−*π*)) = −6.83–0.74 ⋅ Score S_1_+7.11 ⋅ Score N_1_−43.1 ⋅ Score S_2_−68.63 ⋅ Score N_2_−105.01 ⋅ Score S_3_−138.05 ⋅ Score N_3_−105.51 ⋅ Score S_4_−21.16 ⋅ Score N_4_+1.04 ⋅ Score S_5_+81.75 ⋅ Score N_5_−51.83 ⋅ Score S_6_+89.45 ⋅ Score N_6_+5.9 ⋅ Score S_7_−233.07 ⋅ Score N_7_−35.4 ⋅ Score S_8_−20.88 ⋅ Score N_8_. The sensitivity, specificity and accuracy of this model are 0.927, 0.989 and 0.96 respectively. Based on the final logistic regression model, the *π* value of each IAV in Figures 13, 14 and 15 was computed using the following formula: *π* = e*^Y^*/(1+e*^Y^*).

## Supporting Information

Table S1 **List of 96 ORN sequences and their corresponding references.**
(DOC)

Table S2 **List of 2947 non-high pathogenic IAVs.**
(XLS)

Table S3 **List of 3180 non-high pathogenic IAVs.**
(XLS)

Table S4 **The 44 models pass the H-L test from10 fold cross validation repeat 100 times.**
(DOC)
