# Supplementary material for: A Comparative Study of Human TLR 7/8 Stimulatory Trimer Compositions in Influenza A Viral Genomes
Source: PLoS One. 2012 Feb 17;7(2):e30751. doi: 10.1371/journal.pone.0030751 (PMC3281872; doi:10.1371/journal.pone.0030751)
Supplement: Table S1 — List of 96 ORN sequences and their corresponding references. (DOC) [file pone.0030751.s001.doc]

Table S1. Sequences of human TLR 7/8 stimulatory ORNs from research papers.

| Name | Sequence | Cell | Cytokine | PubMed ID |
| --- | --- | --- | --- | --- |
| 9.3 antisense | UGGUAAUUGAAGGACAGGU | Monocyte | IL-12p70 | 19454678 |
| 9.2 antisense | UUGAAGGACAGGUUAAGCU | Monocyte | IL-12p70 | 19454678 |
| 9.3 sense | ACCUGUCCUUCAAUUACCA | Monocyte | IL-12p70 | 19454678 |
| 9.1 antisense | UAAUAACAGUUGCCGUCCA | Monocyte | IL-12p70 | 19454678 |
| 9.2 sense | AGCUUAACCUGUCCUUCAA | Monocyte | IL-12p70 | 19454678 |
| 27+2 sense | GCUGACCCUGAAGUUCAUCUGCACCACUU | pDC | IFNa | 19454678 |
| 27+2 antisense | GUGGUGCAGAUGAACUUCAGGGUCAGCUU | pDC | IFNa | 19454678 |
| 27+0 sense | AAGCUGACCCUGAAGUUCAUCUGCACC | pDC | IFNa | 19454678 |
| 27+0 antisense | GGUGCAGAUGAACUUCAGGGUCAGCUU | pDC | IFNa | 19454678 |
| RNA40 | GCCCGUCUGUUGUGUGACUC | PBMC | TNFa, IL-12p40, IFNa | 14976262 |
| RNA42 | ACCCAUCUAUUAUAUAACUC | PBMC | TNFa, IL-12p40 | 14976262 |
| RNA9.2s | AGCUUAACCUGUCCUUCAATT | pDC | IFNa | 15723075 |
| Influenza virus | A AUAAUUGACCUGCUUUCGCU | PBMC | TNFa, IL-12p40, IFNa | 18072859 |
| RSV | UUGUACGCAUUUUUUCGCGU | PBMC | TNFa | 18072859 |
| Measles virus | CUUACCCAACUUUGUUUGGU | PBMC | TNFa | 18072859 |
| Sendai virus | UGUUUUUUCUCUUGUUUGGU | PBMC | TNFa, IFNa | 18072859 |
| Rabies virus | UUGAUCUGGUUGUUAAGCGU | PBMC | TNFa | 18072859 |
| VSV | AAUGGUUUGUUUGUCUUCGU | PBMC | TNFa | 18072859 |
| 27S | GUCCGGGCAGGUCUACUUUTT | Monocyte | TNFa | 17725606 |
| B-406-AS | UAAUUCGCGUCUGGCCUUCUU | PBMC | TNFa, IFNa | 18250417 |
| Lamin-AS | UGUUCUUCUGGAAGUCCAG | PBMC | TNFa, IFNa | 18250417 |
| si9.2-S | AGCUUAACCUGUCCUUCAA | PBMC | IFNa | 18250417 |
| STAT-2AS | GUUCCAUUGGCUCUGGUGCUU | PBMC | TNFa, IFNa | 18250417 |
| GFP21-AS | GAUGAACUUCAGGGUCAGCUU | PBMC | IFNa | 18250417 |
| SA | GAAGGCCUUACGCGAAUUAUU | PBMC | TNFa | 18250417 |
| SB | GAAGGCCUUACGCGAACAAUU | PBMC | TNFa | 18250417 |
| 1170 | GGACUGCGUUCGCGCUUUCC | PBMC | TNFa, IFNa | 16330816 |
| 1171 | GGCUUAUCCAUUGCACUCCGGA | PBMC | TNFa, IFNa | 16330816 |
| 1172 | GACUAGCUUGCUGUUU | PBMC | TNFa, IFNa | 16330816 |
| 1174 | UUUGUGGUAGUGGGGGACUG | PBMC | TNFa, IFNa | 16330816 |
| 1176 | ACGAAGGUGGUUUUCCCAG | PBMC | TNFa, IFNa | 16330816 |
| 1274 | GGACUGCGUUGUGGCUUUCC | PBMC | TNFa, IFNa | 16330816 |
| 1300 | GAUACUUACCUG | PBMC | TNFa, IFNa | 16330816 |
| 1330 | AAUUUUUGA | PBMC | TNFa, IFNa | 16330816 |
| 1332 | AAUUUGUGG | PBMC | TNFa, IFNa | 16330816 |
| 1337 | GUAGUGUUUGUGGGGGACUG | PBMC | TNFa, IFNa | 16330816 |
| 1338 | GUAGUGGGGGACUGUUUGUG | PBMC | TNFa, IFNa | 16330816 |
| 1563 | GACUAGCCUUU | PBMC | TNFa, IFNa | 16330816 |
| 1-as | AAUUUUGAGAAGAUGAUC | PBMC | TNFa | 16609928 |
| 2-s | GACUUGAGCGAGCGCUUUU | PBMC | TNFa | 16609928 |
| 3-s | GUCCGGGCAGGUCUACUUU | PBMC | TNFa, IFNa | 16609928 |
| 4-s | CCAUCGGAUUGUUCUUUCU | PBMC | TNFa | 16609928 |
| 5-as | UGCUAUUGGUGAUUGCCUC | PBMC | TNFa, IFNa | 16609928 |
| R-1075 | CCGUCUGUUGUGUGACUC | PBMC | TNFa, IFNa | 18322178 |
| R-0001 | UAUAUAUAUAUAUAUAUAUA | PBMC | TNFa | 18322178 |
| R-0002 | UUAUUAUUAUUAUUAUUAUU | PBMC | TNFa | 18322178 |
| R-0003 | UUUAUUUAUUUAUUUAUUUA | PBMC | TNFa, IFNa | 18322178 |
| R-0004 | UUUUAUUUUAUUUUAUUUUA | PBMC | TNFa, IFNa | 18322178 |
| R-0005 | UGUGUGUGUGUGUGUGUGUG | PBMC | TNFa, IFNa | 18322178 |
| R-0006 | UUGUUGUUGUUGUUGUUGUU | PBMC | TNFa, IFNa | 18322178 |
| R-0007 | UUUGUUUGUUUGUUUGUUUG | PBMC | TNFa, IFNa | 18322178 |
| R-0008 | UUUUGUUUUGUUUUGUUUUG | PBMC | TNFa, IFNa | 18322178 |
| R-1312 | GCCACCGAGCGUUGUGUACC | PBMC | TNFa, IFNa | 18322178 |
| R-1321 | GCCACCGAGCAUUGUGAACC | PBMC | TNFa, IFNa | 18322178 |
| R-1322 | GCCACCGAGCAUUGAGAACC | PBMC | TNFa, IFNa | 18322178 |
| R-1323 | GCCACCGAGCAAUGAGAACC | PBMC | TNFa | 18322178 |
| R-1324 | GCCACCGAGCAAGGUGAACC | PBMC | TNFa | 18322178 |
|  | UUGU | PBMC | TNFa, IFNa | 18322178 |
|  | UUUC | PBMC | TNFa, IFNa | 18322178 |
|  | UGUU | PBMC | TNFa, IFNa | 18322178 |
|  | CUGU | PBMC | TNFa, IFNa | 18322178 |
|  | UAUU | PBMC | TNFa, IFNa | 18322178 |
|  | UUUT | PBMC | TNFa, IFNa | 18322178 |
|  | TUGU | PBMC | TNFa, IFNa | 18322178 |
|  | GUUU | PBMC | TNFa, IFNa | 18322178 |
|  | AUUU | PBMC | TNFa, IFNa | 18322178 |
|  | AUGU | PBMC | TNFa, IFNa | 18322178 |
|  | CUUU | PBMC | TNFa, IFNa | 18322178 |
|  | UUUA | PBMC | TNFa, IFNa | 18322178 |
|  | UUGC | PBMC | TNFa, IFNa | 18322178 |
|  | UCUU | PBMC | TNFa, IFNa | 18322178 |
|  | TUUC | PBMC | TNFa, IFNa | 18322178 |
|  | AUUC | PBMC | TNFa, IFNa | 18322178 |
|  | UUCT | PBMC | TNFa, IFNa | 18322178 |
|  | UCUC | PBMC | TNFa, IFNa | 18322178 |
|  | TUUU | PBMC | TNFa, IFNa | 18322178 |
|  | UUGA | PBMC | TNFa, IFNa | 18322178 |
|  | UAUA | PBMC | TNFa, IFNa | 18322178 |
|  | GUUC | PBMC | TNFa, IFNa | 18322178 |
|  | AUAU | PBMC | TNFa, IFNa | 18322178 |
|  | AUAC | PBMC | TNFa, IFNa | 18322178 |
|  | UAUC | PBMC | TNFa, IFNa | 18322178 |
|  | GCUC | PBMC | TNFa, IFNa | 18322178 |
|  | CUAC | PBMC | TNFa, IFNa | 18322178 |
|  | GUAC | PBMC | TNFa, IFNa | 18322178 |
|  | GUGC | PBMC | TNFa, IFNa | 18322178 |
|  | CUGC | PBMC | TNFa, IFNa | 18322178 |
|  | UUCU | PBMC | TNFa, IFNa | 18322178 |
|  | AUAA | PBMC | TNFa, IFNa | 18322178 |
|  | CUAA | PBMC | TNFa, IFNa | 18322178 |
|  | UUUU | PBMC | TNFa, IFNa | 18322178 |
|  | AUUA | PBMC | TNFa, IFNa | 18322178 |
|  | UUAU | PBMC | TNFa, IFNa | 18322178 |
|  | CUCC | PBMC | TNFa, IFNa | 18322178 |
|  | AUCC | PBMC | TNFa, IFNa | 18322178 |
|  | GUCC | PBMC | TNFa, IFNa | 18322178 |

**References:**

1. Ablasser A, Poeck H, Anz D, Berger M, Schlee M, Kim S, Bourquin C, Goutagny N, Jiang Z, Fitzgerald KA, Rothenfusser S, Endres S, Hartmann G, Hornung V. Selection of molecular structure and delivery of RNA oligonucleotides to activate TLR7 versus TLR8 and to induce high amounts of IL-12p70 in primary human monocytes. J Immunol. 2009 Jun 1;182(11):6824-6833.

PMID: 19454678

2. Heil F, Hemmi H, Hochrein H, Ampenberger F, Kirschning C, Akira S, Lipford G, Wagner H, Bauer S. Species-specific recognition of single-stranded RNA via toll-like receptor 7 and 8. Science. 2004 Mar 5;303(5663):1526-1529.

PMID: 14976262

3. Hornung V, Guenthner-Biller M, Bourquin C, Ablasser A, Schlee M, Uematsu S, Noronha A, Manoharan M, Akira S, de Fougerolles A, Endres S, Hartmann G. Sequence-specific potent induction of IFN-alpha by short interfering RNA in plasmacytoid dendritic cells through TLR7. Nat Med. 2005 Mar;11(3):263-270.

PMID: 15723075

4. Forsbach A, Nemorin JG, Völp K, Samulowitz U, Montino C, Müller C, Tluk S, Hamm S, Bauer S, Lipford GB, Vollmer J. Characterization of conserved viral leader RNA sequences that stimulate innate immunity through TLRs. Oligonucleotides. 2007 Winter;17(4):405-417.

PMID: 18072859

5. Furset G, Fløisand Y, Sioud M. Impaired expression of indoleamine 2, 3-dioxygenase in monocyte-derived dendritic cells in response to Toll-like receptor-7/8 ligands. Immunology. 2008 Feb;123(2):263-271.

PMID: 17725606

6. Gantier MP, Tong S, Behlke MA, Xu D, Phipps S, Foster PS, Williams BR. TLR7 is involved in sequence-specific sensing of single-stranded RNAs in human macrophages. J Immunol. 2008 Feb 15;180(4):2117-2124.

PMID: 18250417

7. Vollmer J, Tluk S, Schmitz C, Hamm S, Jurk M, Forsbach A, Akira S, Kelly KM, Reeves WH, Bauer S, Krieg AM. Immune stimulation mediated by autoantigen binding sites within small nuclear RNAs involves Toll-like receptors 7 and 8. J Exp Med. 2005 Dec 5;202(11):1575-1585.

PMID: 16330816

8. Sioud M. Single-stranded small interfering RNA are more immunostimulatory than their double-stranded counterparts: a central role for 2'-hydroxyl uridines in immune responses. Eur J Immunol. 2006 May;36(5):1222-1230.

PMID: 16609928

9. Forsbach A, Nemorin JG, Montino C, Müller C, Samulowitz U, Vicari AP, Jurk M, Mutwiri GK, Krieg AM, Lipford GB, Vollmer J. Identification of RNA sequence motifs stimulating sequence-specific TLR8-dependent immune responses. J Immunol. 2008 Mar 15;180(6):3729-3738.

PMID: 18322178
