# Supplementary material for: A Comparative Study of Human TLR 7/8 Stimulatory Trimer Compositions in Influenza A Viral Genomes
Source: PLoS One. 2012 Feb 17;7(2):e30751. doi: 10.1371/journal.pone.0030751 (PMC3281872; doi:10.1371/journal.pone.0030751)
Supplement: Table S4 — The 44 models pass the H-L test from10 fold cross validation repeat 100 times. (DOC) [file pone.0030751.s004.doc]

Table S4. The 44 models out of 100 iterations which pass the H-L test. Page 4 lists the average of the estimator of *β_1_*, *β_2_* …*β_16_* of these 44 models.

| **Obs** | **Intercept** | **SEG1S** | **SEG1N** | **SEG2S** | **SEG2N** | **SEG3S** | **SEG3N** | **SEG4S** | **SEG4N** | **SEG5S** | **SEG5N** | **SEG6S** |
| --- | --- | --- | --- | --- | --- | --- | --- | --- | --- | --- | --- | --- |
| **1** | -31.3581 | -1.0479 | -11.441 | -64.651 | -68.738 | -106.09 | -140.11 | -100.70 | -21.948 | -0.0265 | 81.966 | -49.320 |
| **2** | -12.6173 | 13.991 | 10.2434 | -41.348 | -67.437 | -108.75 | -133.35 | -99.816 | 2.0579 | -10.616 | 81.403 | -52.274 |
| **3** | -4.4854 | 5.8073 | 4.8082 | -46.470 | -68.932 | -104.47 | -124.46 | -98.976 | -17.140 | 3.5820 | 82.195 | -52.429 |
| **4** | 10.6780 | -4.7152 | 22.7781 | -38.374 | -65.658 | -96.191 | -133.37 | -107.69 | -15.435 | -5.8534 | 73.510 | -45.390 |
| **5** | -23.4316 | -8.7503 | 3.0927 | -48.490 | -64.786 | -102.63 | -154.79 | -105.26 | -32.365 | 2.9902 | 74.778 | -49.914 |
| **6** | -16.8768 | 3.9465 | 7.8509 | -41.569 | -75.784 | -102.14 | -131.17 | -100.38 | -26.699 | 2.9071 | 82.672 | -53.786 |
| **7** | -20.1435 | -4.4819 | -1.1400 | -42.612 | -76.619 | -106.57 | -141.50 | -108.89 | -29.585 | 2.3680 | 86.647 | -51.944 |
| **8** | -27.4569 | 0.2751 | 4.9447 | -47.714 | -67.250 | -101.71 | -145.41 | -101.32 | -34.065 | 2.2208 | 72.569 | -53.426 |
| **9** | 2.5783 | 1.5729 | 17.5528 | -38.853 | -62.444 | -95.937 | -139.81 | -106.65 | -20.580 | 5.5840 | 81.072 | -54.712 |
| **10** | 4.5097 | -4.2348 | 15.3580 | -40.397 | -62.843 | -101.29 | -129.10 | -104.15 | -18.776 | -5.4906 | 77.254 | -49.215 |
| **11** | -19.4175 | 0.7344 | 6.1144 | -49.269 | -77.478 | -108.99 | -136.74 | -104.10 | -26.970 | 0.0023 | 70.972 | -50.004 |
| **12** | -21.3327 | -5.8628 | 12.9206 | -27.434 | -76.432 | -98.759 | -149.78 | -109.05 | -18.494 | 1.3152 | 87.682 | -50.375 |
| **13** | -17.2202 | -8.5221 | 2.4948 | -42.473 | -75.970 | -105.52 | -146.19 | -108.45 | -28.382 | 5.7243 | 84.292 | -49.589 |
| **14** | 37.1054 | -0.8674 | 12.9185 | -29.646 | -70.625 | -106.59 | -122.71 | -108.81 | -4.7558 | 0.6775 | 93.335 | -49.920 |
| **15** | -8.4518 | 11.277 | 14.6354 | -45.307 | -57.221 | -97.834 | -154.26 | -102.51 | -30.942 | 5.9106 | 75.260 | -55.001 |
| **16** | -2.3891 | -0.1250 | 12.8871 | -44.041 | -61.813 | -93.341 | -137.80 | -101.27 | -17.756 | 7.3092 | 81.740 | -51.834 |
| **17** | -0.7379 | -8.4398 | 5.2783 | -50.111 | -62.998 | -101.60 | -143.01 | -105.05 | -27.321 | 6.8437 | 84.728 | -48.525 |
| **18** | 6.7375 | -6.1590 | 20.3018 | -42.024 | -71.502 | -109.39 | -130.66 | -115.05 | -24.809 | -4.7941 | 73.680 | -50.120 |
| **19** | -21.3884 | -5.3011 | 5.7668 | -41.918 | -68.199 | -95.691 | -136.38 | -103.74 | -30.505 | 5.5900 | 81.713 | -53.713 |
| **20** | -7.5623 | -4.5550 | -6.6124 | -48.137 | -65.141 | -110.93 | -138.36 | -104.35 | -34.425 | 2.0106 | 86.186 | -56.650 |
| **21** | 8.0074 | -9.9617 | 10.9076 | -40.032 | -66.188 | -105.53 | -129.53 | -108.14 | -9.9054 | -9.4878 | 81.664 | -47.348 |
| **22** | -19.1280 | -0.9838 | 3.1007 | -44.682 | -71.198 | -103.54 | -144.78 | -107.54 | -22.325 | 7.1345 | 79.139 | -52.696 |
| **23** | -15.2959 | 1.1344 | 0.1205 | -44.103 | -65.453 | -104.66 | -134.22 | -103.74 | -19.461 | -2.5568 | 85.854 | -54.655 |
| **24** | 6.4597 | 2.7086 | 16.0764 | -35.733 | -66.690 | -106.10 | -131.17 | -107.10 | -7.8991 | -6.8719 | 77.935 | -50.376 |
| **25** | 9.5502 | -6.5633 | 5.5459 | -31.343 | -68.136 | -99.334 | -130.39 | -112.08 | -25.340 | 6.5913 | 91.164 | -57.241 |
| **26** | -19.9428 | 3.8247 | -2.5510 | -48.065 | -85.116 | -118.92 | -149.12 | -107.90 | -20.413 | 3.1094 | 86.652 | -49.523 |
| **27** | 1.0168 | 3.9968 | 13.9927 | -44.031 | -61.367 | -101.41 | -139.16 | -104.09 | -25.764 | 10.2495 | 76.209 | -53.259 |
| **28** | -31.3635 | -3.5464 | 5.0923 | -42.737 | -76.352 | -106.34 | -145.10 | -108.57 | -20.880 | 8.1357 | 72.517 | -52.986 |
| **29** | -6.6982 | 1.4989 | 1.1136 | -49.934 | -60.130 | -100.14 | -141.46 | -100.11 | -21.331 | 3.2083 | 87.724 | -50.787 |
| **30** | -11.7544 | 1.7949 | 11.1369 | -39.001 | -65.145 | -109.99 | -129.60 | -107.42 | -21.260 | -7.7040 | 78.410 | -59.209 |
| **31** | -2.5899 | -0.5941 | -2.1692 | -41.991 | -71.631 | -112.67 | -138.39 | -105.37 | -19.983 | 1.7945 | 88.379 | -52.977 |
| **32** | 10.4134 | 6.2730 | -3.5511 | -52.116 | -65.308 | -117.31 | -137.47 | -103.87 | -20.366 | 6.9938 | 86.192 | -54.648 |
| **33** | -28.8852 | 3.8362 | 7.2053 | -48.197 | -73.802 | -110.66 | -140.13 | -99.989 | -12.598 | -7.9874 | 73.684 | -48.070 |
| **34** | -9.7123 | -4.7514 | 0.0873 | -48.582 | -73.284 | -101.74 | -137.28 | -100.97 | -25.668 | 1.3881 | 82.868 | -47.589 |
| **35** | -0.9069 | 1.2562 | 0.9986 | -46.607 | -61.593 | -102.41 | -137.96 | -100.47 | -20.190 | 3.3437 | 85.780 | -53.875 |
| **36** | 29.5519 | 7.5958 | 27.5741 | -28.240 | -65.115 | -116.68 | -122.33 | -106.87 | 1.3437 | -17.551 | 79.798 | -48.253 |
| **37** | -8.2696 | -8.0948 | -7.4131 | -48.052 | -75.631 | -112.81 | -132.34 | -109.15 | -20.196 | 0.6375 | 92.540 | -51.910 |
| **38** | -11.8969 | -3.0745 | -2.2582 | -49.752 | -65.902 | -107.48 | -143.85 | -105.74 | -25.082 | 10.3720 | 84.834 | -53.077 |
| **39** | 8.4828 | -8.7030 | 14.0646 | -32.639 | -66.913 | -110.78 | -147.18 | -108.44 | -4.4154 | -7.7859 | 82.563 | -44.148 |
| **40** | -22.0644 | 4.2112 | 19.1898 | -42.563 | -76.892 | -105.20 | -142.67 | -117.28 | -40.132 | -4.3276 | 82.268 | -55.127 |
| **41** | 1.1857 | 0.6178 | 9.7376 | -44.263 | -64.499 | -108.69 | -135.74 | -106.22 | -18.578 | 4.8552 | 75.879 | -53.592 |
| **42** | -27.0470 | -2.3026 | 5.7725 | -39.232 | -74.053 | -98.045 | -136.80 | -104.02 | -25.039 | 3.7827 | 85.322 | -54.625 |
| **43** | -0.1122 | 2.7836 | 9.4931 | -41.661 | -63.943 | -104.59 | -143.22 | -106.10 | -22.005 | 4.1975 | 78.777 | -55.418 |
| **44** | 13.6569 | -0.1982 | 8.7861 | -42.214 | -67.685 | -101.09 | -135.26 | -104.95 | -24.737 | 5.8990 | 87.246 | -51.175 |

| **Obs** | **SEG6N** | **SEG7S** | **SEG7N** | **SEG8S** | **SEG8N** | **ProbChiSq** | **prec** | **acc** | **sp** | **se** | **err_rate** |
| --- | --- | --- | --- | --- | --- | --- | --- | --- | --- | --- | --- |
| **1** | 88.624 | 13.143 | -250.53 | -40.891 | -24.476 | 0.0879 | 0.9117 | 0.9535 | 0.9252 | 0.9897 | 0.04655 |
| **2** | 78.752 | 9.6031 | -251.08 | -39.8851 | -25.054 | 0.0675 | 0.9105 | 0.9535 | 0.9214 | 0.9933 | 0.04655 |
| **3** | 82.710 | -2.1707 | -235.55 | -34.325 | -18.894 | 0.1346 | 0.9167 | 0.9550 | 0.9214 | 0.9936 | 0.04505 |
| **4** | 84.467 | 7.6355 | -218.73 | -34.414 | -22.146 | 0.0800 | 0.9100 | 0.9535 | 0.9310 | 0.9856 | 0.04655 |
| **5** | 94.628 | 1.7305 | -232.74 | -36.521 | -21.689 | 0.0629 | 0.9233 | 0.9580 | 0.9357 | 0.9864 | 0.04204 |
| **6** | 82.385 | 3.6582 | -224.08 | -34.094 | -18.368 | 0.1240 | 0.9200 | 0.9520 | 0.9280 | 0.9803 | 0.04805 |
| **7** | 93.483 | 7.8162 | -235.81 | -36.619 | -20.341 | 0.1590 | 0.9231 | 0.9565 | 0.9357 | 0.9829 | 0.04354 |
| **8** | 96.374 | 2.1463 | -233.24 | -36.251 | -22.351 | 0.1060 | 0.9290 | 0.9595 | 0.9363 | 0.9869 | 0.04054 |
| **9** | 91.480 | 4.7387 | -227.53 | -34.068 | -17.570 | 0.0545 | 0.9003 | 0.9475 | 0.9189 | 0.9859 | 0.05255 |
| **10** | 86.418 | 4.1235 | -227.89 | -34.748 | -30.700 | 0.2547 | 0.9174 | 0.9535 | 0.9205 | 0.9905 | 0.04655 |
| **11** | 90.930 | 3.9557 | -230.23 | -35.844 | -23.126 | 0.1393 | 0.9219 | 0.9565 | 0.9319 | 0.9866 | 0.04354 |
| **12** | 90.845 | 2.2884 | -230.85 | -33.743 | -23.822 | 0.0612 | 0.8994 | 0.9444 | 0.9101 | 0.9866 | 0.05556 |
| **13** | 91.100 | 5.4885 | -228.26 | -34.577 | -18.070 | 0.1733 | 0.9223 | 0.9581 | 0.9364 | 0.9862 | 0.04204 |
| **14** | 89.189 | 11.989 | -220.81 | -37.122 | -15.915 | 0.0681 | 0.9317 | 0.9565 | 0.9387 | 0.9772 | 0.04354 |
| **15** | 104.88 | 6.4883 | -227.41 | -37.384 | -12.468 | 0.0877 | 0.9284 | 0.9565 | 0.9365 | 0.9803 | 0.04354 |
| **16** | 82.013 | -2.4026 | -225.96 | -31.545 | -15.624 | 0.0700 | 0.9000 | 0.9490 | 0.9149 | 0.9931 | 0.05105 |
| **17** | 89.052 | 1.6311 | -225.43 | -32.113 | -19.298 | 0.0896 | 0.9049 | 0.9520 | 0.9251 | 0.9893 | 0.04805 |
| **18** | 82.339 | 8.3918 | -226.49 | -30.518 | -24.362 | 0.1492 | 0.9061 | 0.9535 | 0.9288 | 0.9890 | 0.04655 |
| **19** | 88.666 | 1.4010 | -230.89 | -35.449 | -25.046 | 0.1556 | 0.9203 | 0.9490 | 0.9274 | 0.9740 | 0.05105 |
| **20** | 100.14 | 10.766 | -231.13 | -34.094 | -25.673 | 0.0552 | 0.9191 | 0.9565 | 0.9339 | 0.9861 | 0.04354 |
| **21** | 77.247 | 9.9711 | -232.42 | -36.438 | -28.790 | 0.1057 | 0.9099 | 0.9505 | 0.9167 | 0.9902 | 0.04955 |
| **22** | 86.161 | 3.6769 | -240.86 | -39.112 | -10.029 | 0.1160 | 0.9252 | 0.9535 | 0.9288 | 0.9810 | 0.04655 |
| **23** | 87.259 | 14.531 | -243.50 | -40.306 | -23.909 | 0.1368 | 0.9167 | 0.9550 | 0.9352 | 0.9821 | 0.04505 |
| **24** | 86.193 | 8.7355 | -234.28 | -36.115 | -24.414 | 0.0832 | 0.9348 | 0.9640 | 0.9420 | 0.9901 | 0.03604 |
| **25** | 88.721 | 12.738 | -227.93 | -37.551 | -9.5956 | 0.1572 | 0.8962 | 0.9444 | 0.9125 | 0.9862 | 0.05556 |
| **26** | 98.172 | 5.2776 | -239.63 | -36.877 | -20.118 | 0.0630 | 0.8903 | 0.9414 | 0.9074 | 0.9861 | 0.05856 |
| **27** | 93.098 | 1.1226 | -226.45 | -32.963 | -12.219 | 0.0530 | 0.9233 | 0.9580 | 0.9309 | 0.9901 | 0.04204 |
| **28** | 83.846 | -2.0157 | -242.95 | -32.002 | -13.184 | 0.0657 | 0.9218 | 0.9595 | 0.9368 | 0.9895 | 0.04054 |
| **29** | 84.739 | 5.4570 | -229.26 | -31.820 | -14.189 | 0.1445 | 0.9174 | 0.9580 | 0.9260 | 0.9967 | 0.04204 |
| **30** | 83.521 | 6.8057 | -248.29 | -35.753 | -31.088 | 0.0611 | 0.8971 | 0.9414 | 0.9158 | 0.9755 | 0.05856 |
| **31** | 100.13 | 11.632 | -233.83 | -35.622 | -22.560 | 0.0908 | 0.9097 | 0.9490 | 0.9214 | 0.9832 | 0.05105 |
| **32** | 106.18 | 5.5774 | -241.06 | -36.956 | -18.417 | 0.0914 | 0.8987 | 0.9475 | 0.9156 | 0.9896 | 0.05255 |
| **33** | 85.778 | 6.8337 | -243.77 | -36.640 | -30.944 | 0.0809 | 0.8918 | 0.9354 | 0.8958 | 0.9807 | 0.06457 |
| **34** | 88.799 | 5.1564 | -222.60 | -36.836 | -19.494 | 0.0681 | 0.9195 | 0.9550 | 0.9288 | 0.9867 | 0.04505 |
| **35** | 94.008 | 3.4296 | -233.57 | -34.387 | -19.351 | 0.0769 | 0.9288 | 0.9625 | 0.9415 | 0.9897 | 0.03754 |
| **36** | 78.013 | 12.676 | -230.93 | -39.825 | -30.461 | 0.0834 | 0.8974 | 0.9475 | 0.9207 | 0.9855 | 0.05255 |
| **37** | 94.377 | 8.3828 | -244.77 | -32.650 | -28.969 | 0.1273 | 0.8679 | 0.9294 | 0.8824 | 0.9897 | 0.07057 |
| **38** | 87.644 | -0.0266 | -240.15 | -33.896 | -11.873 | 0.1067 | 0.8909 | 0.9399 | 0.9022 | 0.9866 | 0.06006 |
| **39** | 88.784 | 3.4410 | -228.93 | -31.933 | -28.551 | 0.0667 | 0.9198 | 0.9538 | 0.9294 | 0.9835 | 0.04620 |
| **40** | 85.453 | 12.882 | -233.65 | -34.523 | -21.681 | 0.1394 | 0.9015 | 0.9429 | 0.9128 | 0.9799 | 0.05707 |
| **41** | 98.089 | 2.6412 | -237.87 | -33.522 | -24.188 | 0.0900 | 0.9216 | 0.9580 | 0.9368 | 0.9860 | 0.04204 |
| **42** | 80.747 | 4.5232 | -233.38 | -33.719 | -19.510 | 0.1099 | 0.9229 | 0.9550 | 0.9259 | 0.9873 | 0.04505 |
| **43** | 97.647 | 3.9958 | -232.32 | -34.847 | -17.355 | 0.0743 | 0.9256 | 0.9565 | 0.9288 | 0.9873 | 0.04354 |
| **44** | 92.573 | 9.7759 | -217.83 | -39.183 | -12.907 | 0.1574 | 0.9144 | 0.9444 | 0.9218 | 0.9708 | 0.05556 |

| **Variable** | **Mean** |
| --- | --- |
| Intercept SEG1S SEG1N SEG2S SEG2N SEG3S SEG3N SEG4S SEG4N SEG5S SEG5N SEG6S SEG6N SEG7S SEG7N SEG8S SEG8N | -6.8318935 -0.7431425 7.1092095 -43.1051792 -68.6343760 -105.016574 -138.052885 -105.511813 -21.1622210 1.0380750 81.7517027 -51.8346584 89.4472308 5.9010462 -233.069352 -35.4022561 -20.8819897 |

yhat = -6.8318935-0.7431425* SEG1S+ 7.1092095* SEG1N -43.1051792* SEG2S

-68.6343760* SEG2N-105.0165740* SEG3S -138.0528853*SEG3N -105.5118134* SEG4S

-21.1622210* SEG4N+1.0380750* SEG5S+ 81.7517027*SEG5N -51.8346584* SEG6S

+89.4472308* SEG6N+5.9010462* SEG7S-233.0693529* SEG7N-35.4022561* SEG8S

-20.8819897* SEG8N;

model_mean 2011年08月24日 星期三 上午06時10分13秒 1

The FREQ Procedure

1:誤判

Cumulative

Cumulative

ct Frequency Percent Frequency

Percent

-------------------------------------------------------

0 6343 95.27 6343

95.27

1 315 4.73 6658

100.00

0->1

Cumulative

Cumulative

e1 Frequency Percent Frequency

Percent

-------------------------------------------------------

0 6381 95.84 6381

95.84

1 277 4.16 6658

100.00

1->0

Cumulative

Cumulative

e2 Frequency Percent Frequency

Percent

-------------------------------------------------------

0 6620 99.43 6620

99.43

1 38 0.57 6658

100.00
